# Aquaporin 9 inhibits hepatocellular carcinoma through up-regulating FOXO1 expression

**DOI:** 10.18632/oncotarget.10143

**Published:** 2016-06-17

**Authors:** Chuan-Fei Li, Wen-Guang Zhang, Min Liu, Lie-Wang Qiu, Xiao-Feng Chen, Lin Lv, Zhe-Chuan Mei

**Affiliations:** ^1^ Department of Gastroenterology, The Second Affiliated Hospital, Chongqing Medical University, Chongqing, China; ^2^ Department of Gastroenterology, Yongchuan Hospital, Chongqing Medical University, Chongqing, China; ^3^ The First Branch of The First Affiliated Hospital, Chongqing Medical University, Chongqing, China

**Keywords:** liver cancer, aquaporin 9, cell cycle, apoptosis, FOXO1

## Abstract

Aquaporin 9 (AQP9) is the main aquaglyceroporin in the liver. Few studies have been performed regarding the role of AQP9 in liver cancer. Here we report AQP9 expression and function in liver cancer. We found that AQP9 mRNA and protein levels were reduced in human hepatocellular cancer compared to the para-tumor normal liver tissues. Human hepatoma cell line SMMC7721 expressed low basal levels of AQP9. When AQP9 was overexpressed in SMMC7721 cell line, cell proliferation was inhibited due to cell cycle arrest at G1 phase and increased apoptosis. At the molecular level, AQP9 overexpression decreased the protein levels of phosphatidylinositol-3-kinase (PI3K), leading to reduced phosphorylation of Akt. Subsequently, the protein levels of forkhead box protein O1 (FOXO1) were increased, resulting in down-regulation of proliferating cell nuclear antigen (PCNA) expression and up-regulation of caspase-3 expression. AQP9 overexpression inhibited growth of subcutaneously xenografted liver tumors in nude mice. These findings suggest that AQP9 expression is down-regulated in liver cancer compared to the normal liver tissue and restoration of AQP9 expression can inhibit development of liver cancer.

## INTRODUCTION

Hepatocellular carcinoma (HCC) is the sixth most common malignancy worldwide and is the third leading cause of cancer-related deaths after lung and gastric cancers [1]. Aquaporins (AQPs) are transmembrane proteins that mediate water molecules transporting across the cytoplasmic membrane. Currently, 13 AQP subtypes (AQP0-AQP12) have been identified in mammalian cells [2]. AQPs are divided into three categories: (1) orthodox aquaporins, including AQP1/2/4/5; (2) aquaglyceroporins, including AQP3/7/9/10; and (3) unorthodox aquaporins, including AQP6/8/11/12 [3]. Aquaglyceroporins are not only permeable to water, glycerin, urea and other small solutes, but also mediates transport of 5-fluorouracil and other anticancer drugs [4]. AQPs are widely distributed in human tissues and have been found to be involved in edema [5], glucose and lipid metabolism [6, 7], and skin physiology [8] as well as cell migration [9]. AQPs play different roles in different tissues and organs.

It has been shown that seven AQPs are expressed in the liver, where they play important pathophysiological roles [10]. AQP9 is the most important aquaglyceroporin in the liver and is localized at the basolateral membrane of hepatocytes [11]. Several previous studies on AQP9 focused on its metabolic functions. For example, Rodriguez et al. [7] found that insulin acts through phosphatidylinositol-3-kinase (PI3K)-Akt-mammalian target of rapamycin to control expression of AQP9. Our group reported that sodium oleate can regulate AQP9 expression via p38 mitogen-activated protein kinase [12]. However, few studies have investigated the role of AQP9 in liver cancer. Therefore, the purpose of the present study was to investigate AQP9 expression and function in liver cancer. We found that AQP9 mRNA and protein levels were reduced in human hepatocellular cancer compared to the para-tumor normal liver tissues. When AQP9 was overexpressed in SMMC7721 human hepatoma cell line, cell colony formation was reduced due to cell cycle arrest at G1 phase and increased apoptosis, which was linked to reduced PI3K and P-Akt levels and increased forkhead box protein O1 (FOXO1) levels. AQP9 overexpression inhibited growth of subcutaneously xenografted liver tumors in nude mice. These findings suggest that restoration of AQP9 expression can inhibit tumor growth of liver cancer in nude mice.

## RESULTS

### AQP9 expression is down-regulated in human liver cancer

**Figure 1:**
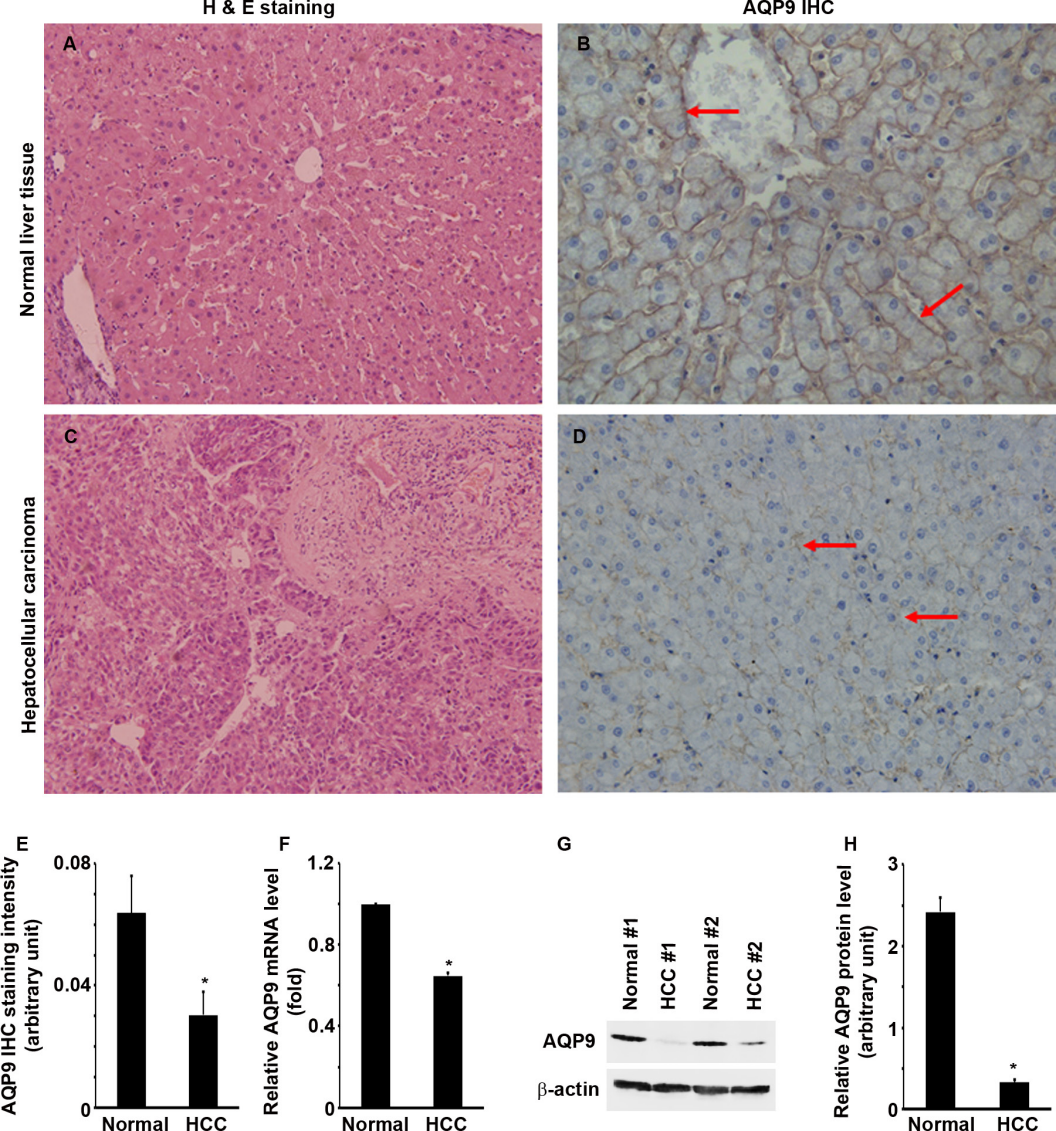
AQP9 expression is down-regulated in human liver cancer (please note that this editing is done in the blot panels in Figure 1G) (**A** and **C**) H&E staining of HCC and human normal liver tissues. (**B** and **D**) AQP9 IHC; arrows indicate the positive cells. Original magnification, ×400. (**E**) Quantification of AQP9 IHC staining; *n* = 34 per group, *P < 0.01. (**F**) Quantitative RT-PCR analysis of AQP9 mRNA expression; *n* = 34 per group, *P < 0.01. (**G**) Representative Western blot analysis of AQP9 expression in 2 pairs of human hepatocellular cancer and the para-tumor normal liver tissues. (**H**) Quantification of Western blot analysis of AQP9 protein levels normalized by the levels of β-actin; *n* = 34 per group, *P < 0.001.

We did IHC staining of AQP9 in 34 cases of human HCC and their matched normal liver tissues. Pathologic diagnosis of normal liver tissues and HCC was confirmed by hematoxin and eosin (H&E) staining (Figure 1A and 1C). AQP9 staining was localized at the cytoplasmic membrane of normal hepatocytes, particularly adjacent to the sinusoid and central vein (Figure 1B). However, AQP9 staining was weak or absent in the HCC cells (Figure 1D). AQP9 staining was significantly weaker in HCC than normal liver tissues (Figure 1E, *P* < 0.01). AQP9 mRNA levels were significantly lower in HCC than normal liver tissues (Figure 1F, *P* < 0.01). Further, AQP9 protein levels were also significantly lower in HCC than normal liver tissues (Figure 1G and 1H, *P* < 0.001). Association analysis showed that the decline of AQP9 level was relevant with the stage of tumor, tumor differentiation and tumor metastasis (Supplementary Table S1, *P* < 0.01). There was a positive correlation between expression decrease of AQP9 and the above indexes. Association analysis indicated that it is not correlated to the patients' age, gender, primary liver disease, tumor size and lymph node metastasis (Supplementary Table S1, *P* > 0.05).

### Overexpression of AQP9 in liver cancer cells inhibits cell proliferation

**Figure 2:**
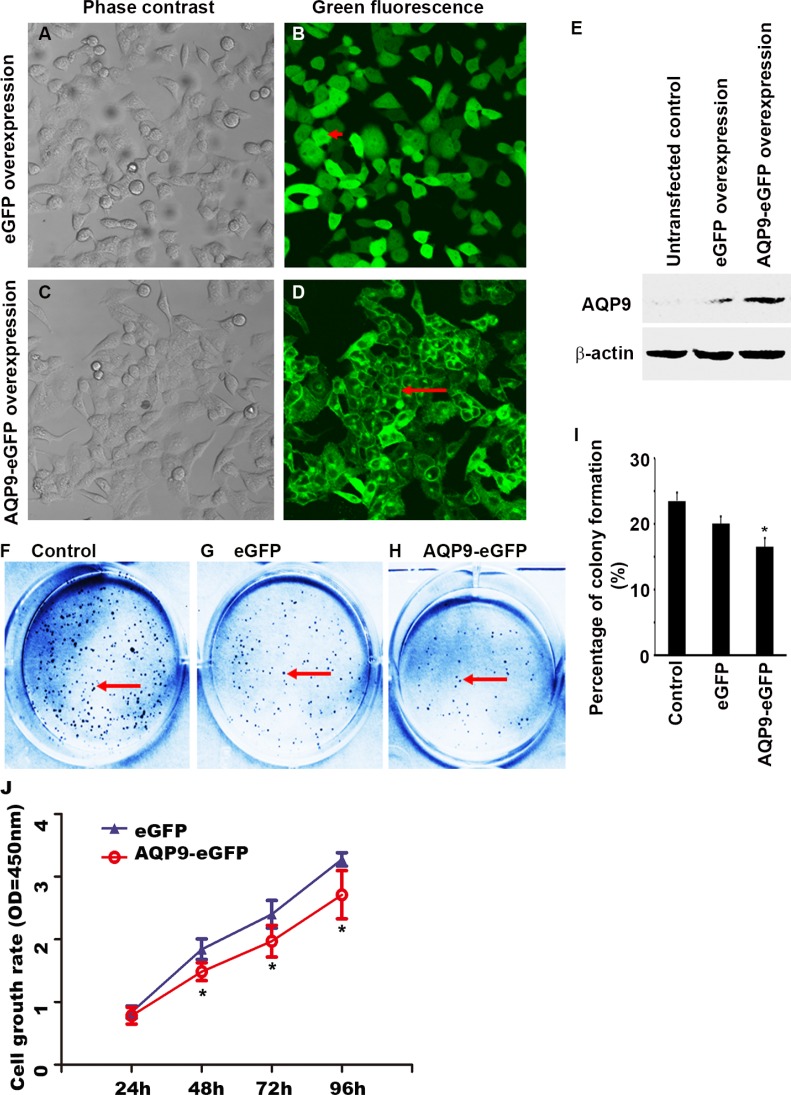
Overexpression of AQP9 in liver cancer cells inhibits cell proliferation (**A**–**D**) SMMC7721 cells were transfected with lentiviruses expressing either eGFP or AQP9-eGFP fusion protein; all cells showed eGFP expression under a fluorescence microscope. (**E**) Expression of AQP9 in the transfected cells was confirmed by Western blot analysis. (**F**–**H**) Representative pictures of colony formation assay showed Giemsa stained cell colonies in the control cells (F), eGFP^*+*^ cells (G), and AQP9-eGFP^*+*^ cells (H). (**I**) Quantification of the colony formation efficiency. (**J**) CCK8 assay showed that after 24 h, 48 h, 72 h, and 96 h of transfection, AQP9 expression significantly inhibited the cell growth rates; *P < 0.05, compared to the eGFP^+^ cells.

SMMC7721 cells were transfected with lentiviruses expressing either eGFP or AQP9-eGFP fusion protein. After puromycin-based selection, all cells expressed eGFP (Figure 2A to 2D). However, the eGFP signals appeared throughout the entire cell in the eGFP^*+*^ cells (Figure 2B), whereas the eGFP signals were localized on the cytoplasmic membrane in the AQP9-eGFP^*+*^ cells (Figure 2D), mimicking the transmembrane localization of endogenous AQP9. The protein levels of AQP9 in the transfected cells were confirmed by Western blot analysis (Figure 2E). The AQP9-eGFP^*+*^ cells formed significantly less colonies than either the eGFP^*+*^ cells or control cells without any transfection (Figure 2F to 2I, *P* < 0.05). The growth of the AQP9-eGFP^*+*^ cells was inhibited after 24 h, 48 h, 72 h, and 96 h of transfection compared to the eGFP+ cells (Figure 2J, *P* < 0.05).

### Overexpression of AQP9 in liver cancer cells induces cell cycle arrest and apoptosis

**Figure 3:**
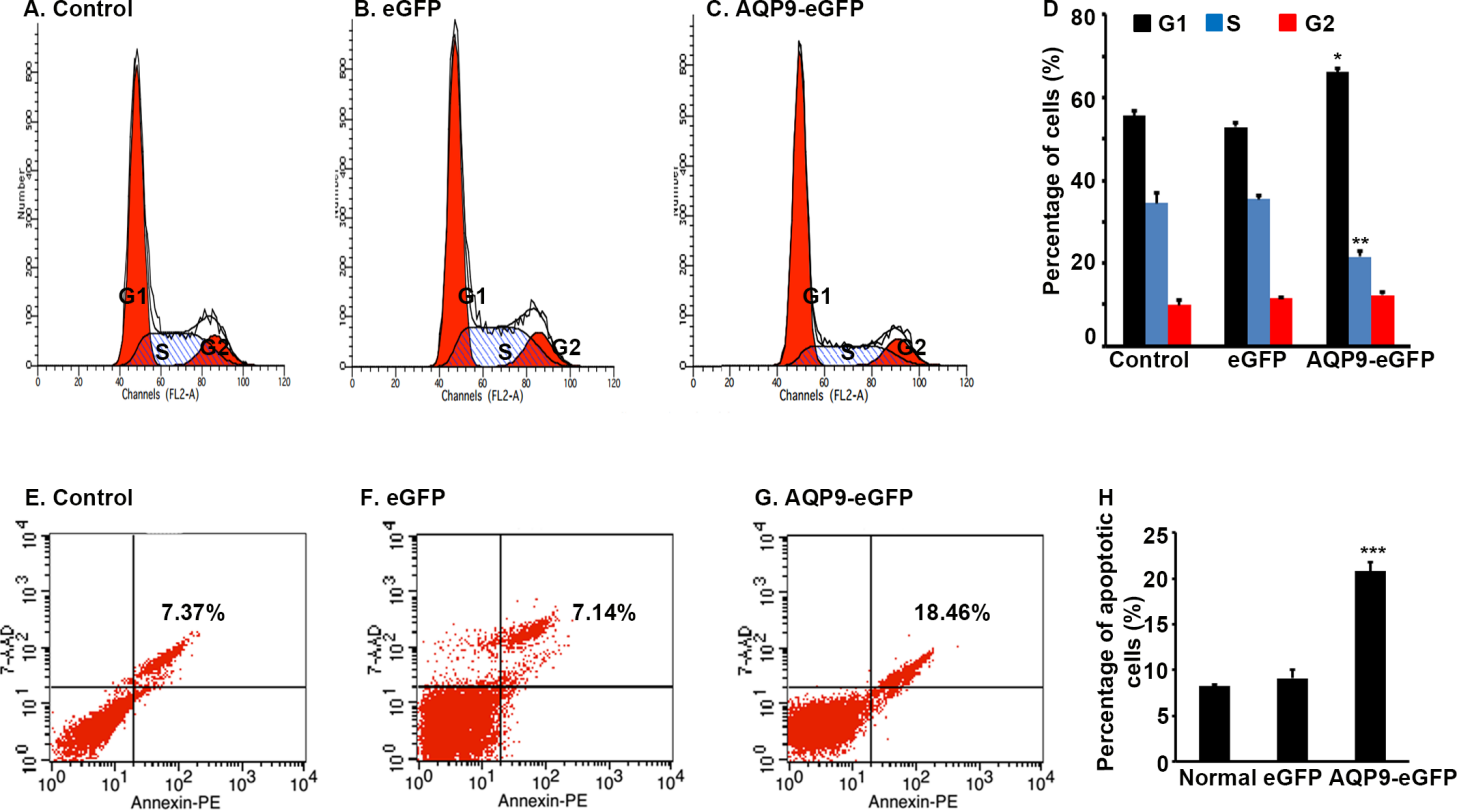
Overexpression of AQP9 in liver cancer cells induces cell cycle arrest and apoptosis (**A**–**C**) Representative histograms of flow cytometry analysis of cell cycle. (**D**) Percentage of cells at the G1, S, and G2/M phase. Data represent the mean ± standard error of the mean of 3 independent experiments; *P < 0.01 and **P < 0.01, compared to the corresponding control cells or eGFP^*+*^ cells. (**E**–**G**) Representative scatter plots of flow cytometry analysis of apoptotic cells. (**H**) Percentage of apoptotic cells. Data represent the mean ± standard error of the mean of 3 independent experiments; ***P < 0.01, compared to the corresponding control cells or eGFP^*+*^ cells.

Flow cytometry analysis showed that the percentage of cells at G1 phase was significantly higher in the AQP9-eGFP^*+*^ cells than the eGFP^*+*^ cells or control cells, while the percentage of cells at S phase was significantly lower in the AQP9-eGFP^*+*^ cells than the eGFP^*+*^ cells or control cells (Figure 3A to 3D, *P* < 0.01). On the other hand, the percentage of apoptotic cells was significantly higher in the AQP9-eGFP^*+*^ cells than the eGFP^*+*^ cells or control cells (Figure 3E to 3H, *P* < 0.01).

### Overexpression of AQP9 in liver cancer cells inhibits PI3K/Akt signaling to increase FOXO1 levels

**Figure 4:**
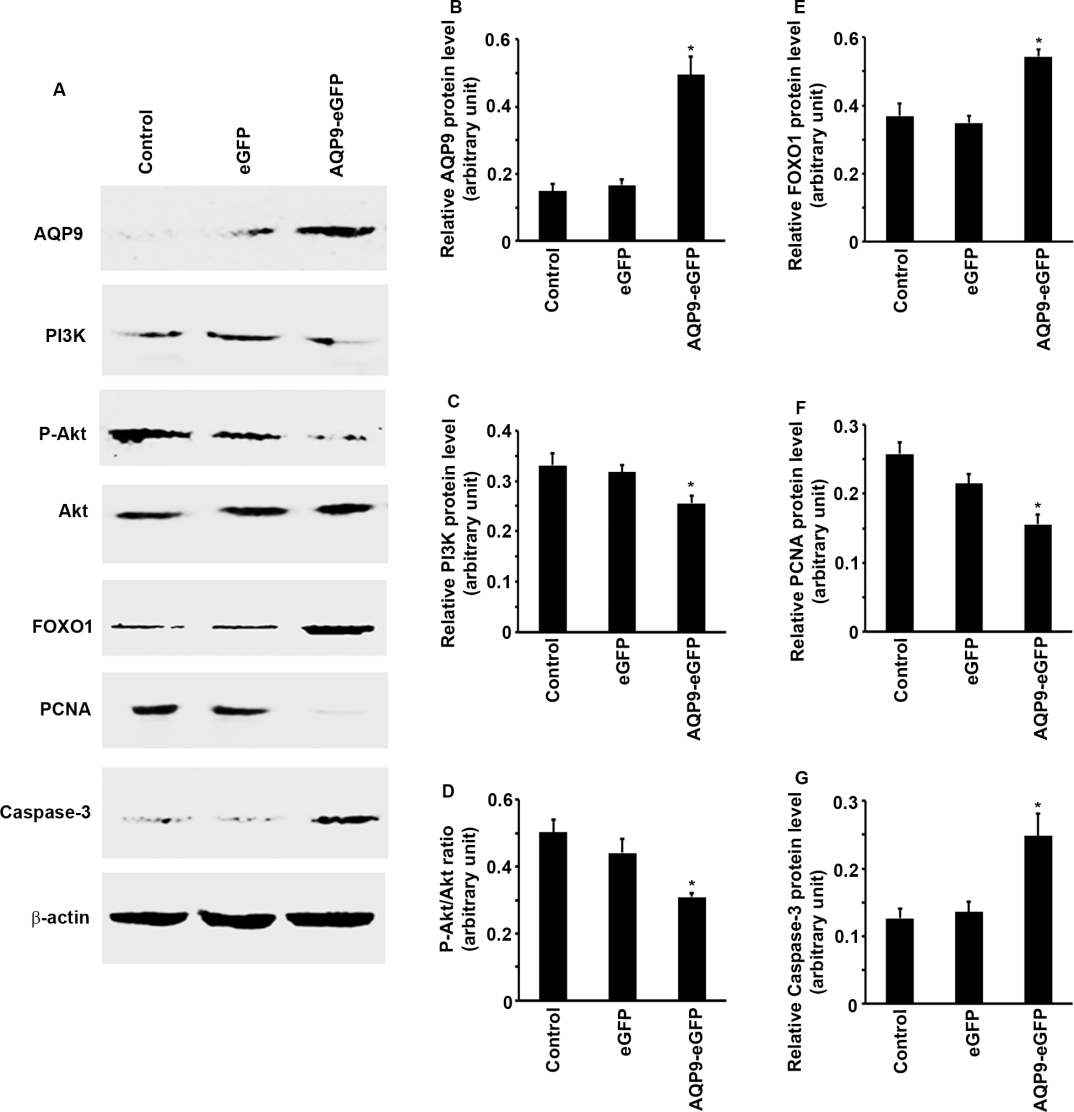
Overexpression of AQP9 in liver cancer cells inhibits PI3K/Akt signaling to increase FOXO1 levels (**A**–**E**) After AQP9 overexpress, expression levels of PI3K and P-Akt were reduced, but the protein levels of FOXO1 were significantly increased, the levels of P-Akt were normalized by the levels of Akt. (**A**, **F** and **G**) After AQP9 overexpress, the protein levels of PCNA were significantly decreased, but the protein levels of caspase-3 were significantly increased. The levels of each indicated protein were normalized by the levels of β-actin. Data represent the mean ± standard error of the mean of 3 independent experiments; *P < 0.05, compared to the corresponding control cells or eGFP+ cells.

To understand the molecular changes caused by AQP9 overexpression in SMMC7721 cells, we examined multiple intracellular signaling pathways. We found that upon AQP9 overexpression, the protein levels of PI3K were significantly reduced in the AQP9-eGFP^*+*^ cells compared to the eGFP^*+*^ cells or control cells (Figure 4A to 4C, *P* < 0.05). Since PI3K is responsible for phosphorylation of Akt, we found that P-Akt levels were also reduced, thus the P-Akt/Akt ratio was significantly reduced in the AQP9-eGFP^*+*^ cells compared to the eGFP^*+*^ cells or control cells (Figure 4A and 4D, *P* < 0.05). The protein levels of FOXO1 were significantly increased in the AQP9-eGFP^*+*^ cells compared to the eGFP^*+*^ cells or control cells (Figure 4A and 4E, *P* < 0.05). Further, the protein levels of PCNA were significantly decreased, but the protein levels of caspase-3 were significantly increased in the AQP9-eGFP^*+*^ cells compared to the eGFP^*+*^ cells or control cells (Figure 4A, 4F, and 4G, *P* < 0.05).

### Overexpression of AQP9 inhibits liver tumor growth in nude mice

**Figure 5:**
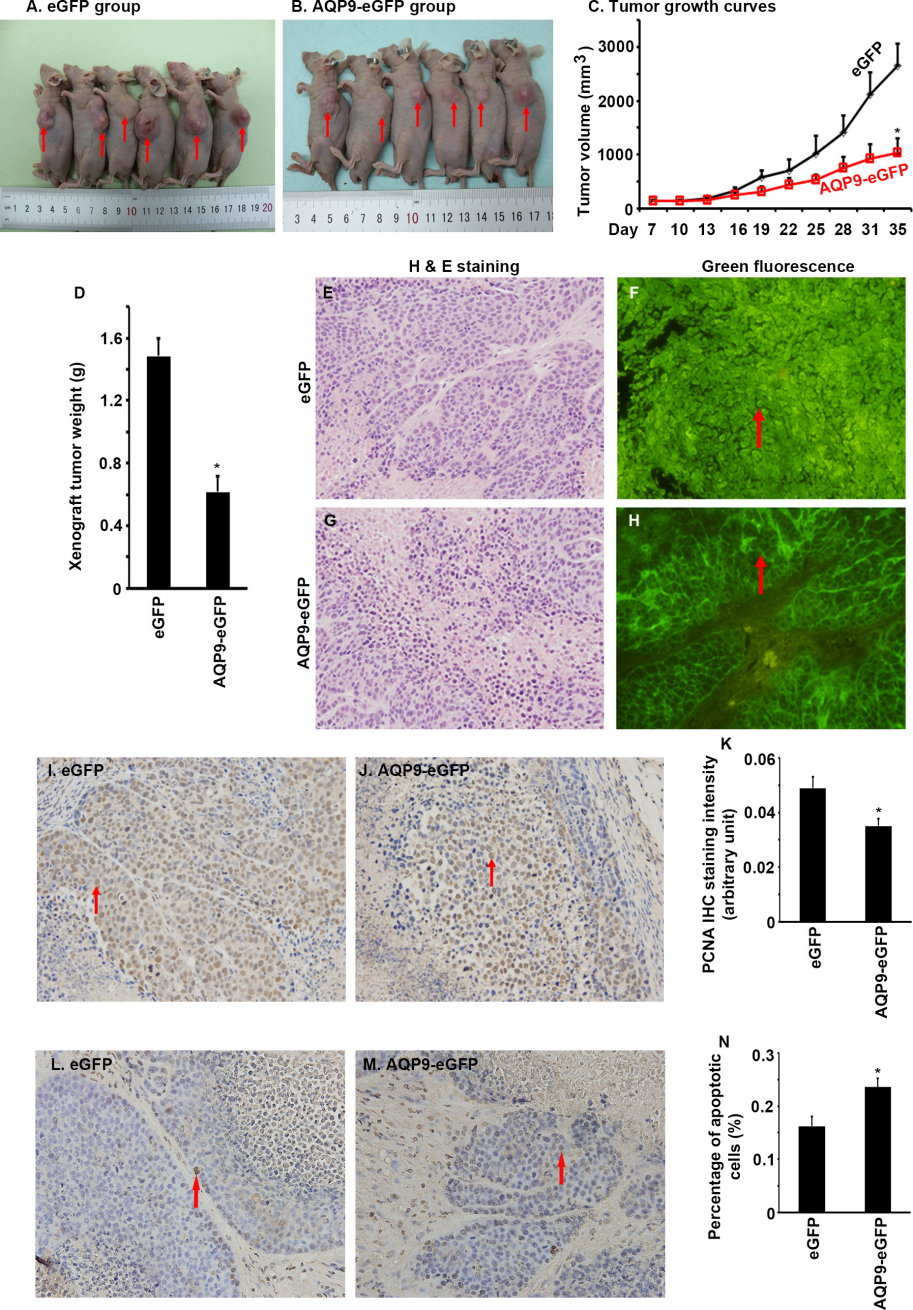
Overexpression of AQP9 inhibits liver tumor growth in nude mice (**A** and **B**) Pictures of euthanized nude mice bearing subcutaneously xenografted liver tumors derived either from the eGFP^+^ cells (A) or the AQP9-eGFP^*+*^ cells (B); arrows indicate the subcutaneous tumors. (**C**) Tumor growth curves; *n* = 6 per group, *P < 0.01. (**D**) Xenograft tumor weight; *n* = 6 per group, *P < 0.01. (**E** to **H**) Histopathological examination of the tumors; arrows indicate the localization of eGFP signals. (**I** and **J**) Representative IHC staining of PCNA; arrows indicate the positive cells; original magnification, ×400. (**K**) Quantification of PCNA IHC staining intensity; *n* = 6 per group, *P < 0.05. (**L** and **M**) Representative photomicrographs of TUNEL assays; arrows indicate the positive cells; original magnification, ×400. (**N**) Percentage of apoptotic cells; *n* = 6 per group, *P < 0.05.

To assess the effects of AQP9 overexpression on liver tumor growth *in vivo*, we injected the AQP9-eGFP^*+*^ cells and eGFP^*+*^ cells subcutaneously in nude mice. We found that the xenografted tumors derived from the AQP9-eGFP^*+*^ cells grew significantly slower than the tumors derived from the eGFP^*+*^ cells (Figure 5A to 5C, *P* < 0.01). The endpoint xenograft tumor weight was significantly lighter in the AQP9-eGFP^*+*^ tumors than the eGFP^*+*^ tumors (Figure 5D, *P* < 0.01). Histopathological examination showed that the tumor cells retained expression of eGFP and the AQP9-eGFP^*+*^ tumor cells kept the cytoplasmic membrane-localization of eGFP (Figure 5E to 5H). IHC staining showed that the AQP9-eGFP^*+*^ tumors had significantly less PCNA staining than the eGFP^*+*^ tumors (Figure 5I to 5K, *P* < 0.05). On the other hand, the AQP9-eGFP^*+*^ tumors had significantly more apoptotic cells than the eGFP^*+*^ tumors (Figure 5L to 5N, *P* < 0.05).

## DISCUSSION

The molecular mechanisms of HCC pathogenesis have not been fully understood. Liver cancer appears as a result of the disrupted balance between cellular proliferation and apoptosis [13]. In the present study, we demonstrated that AQP9 expression was down-regulated in human HCC compared to normal liver tissues, which is consistent with a previous report [14]. What we extended further is that AQP9 overexpression in a human hepatoma cell line inhibited cell growth by causing cell cycle arrest at G1 phase and apoptosis. AQP9 overexpression also inhibited xenografted liver tumor growth in nude mice. It has been reported that activated hepatic stellate cells down-regulated expression of AQPs, thus becoming more resistant to apoptosis [15]. Decreased aquaporin expression also leads to increased resistance to apoptosis in hepatocellular carcinoma [16]. In our study, overexpression of AQP9 made the cancer cells sensitive to apoptosis both *in vitro* and *in vivo* conditions.

How AQP9 overexpression sensitizes cancer cells to apoptosis is not quite clear. We found that in the AQP9-eGFP^*+*^ cells, PI3K protein levels were reduced, which in turn led to reduced phosphorylation of Akt. It is not clear how AQP9 affects PI3K expression, but other AQPs have also been shown to act on PI3K/Akt pathway. Xu et al. [17] reported that overexpression of AQP3 increased phosphorylation of Akt in human gastric carcinoma SGC7901 cells. The discrepancy between this study and ours may be caused by the differences in aquaporin subtypes and the differences in cancer cell lines, indicating aquaporin subtype-specific or cell type-specific actions of AQPs. To support this speculation, it has been reported that AQP1 expression was up-regulated in lung cancer and forced expression of AQP1 in NIH-3T3 cells induced cell proliferation and anchorage-independent growth in soft agar [18]. Nevertheless, it has been well established that Akt phosphorylates FOXO1, leading to nuclear export of FOXO1 and proteasomal degradation of FOXO1 [19, 20, 21, 22]. When PI3K/Akt activities are inhibited by AQP9 overexpression, FOXO1 levels are increased and are functional in the nucleus. FOXO1 has been shown to control expression of pro-apoptotic gene families including caspases [23]. In our study, caspase-3 levels were up-regulated in the AQP9-eGFP^*+*^ cells in accompanying with high levels of FOXO1, which may contribute to the increased apoptosis observed in the AQP9-eGFP^*+*^ cells.

FOXOs including FOXO1 have been known to regulate expression of a variety of cell cycle regulatory genes, such as cyclin A, cyclin B, cyclin D, cyclin E, cyclin G2, p15, p19, p21, p27, and p130 [24]. One of the main actions of FOXOs is to induce cell cycle arrest at the G1/S boundary [25], which was also observed in our study. In the AQP9-eGFP^*+*^ cells, FOXO1 levels were increased, leading to increased number of cells in the G1 phase and reduced number of cells in the S phase. Thus, cellular proliferation was inhibited as shown by reduced levels of PCNA in the cells and tumors when AQP9 was overexpressed.

In conclusion, we demonstrated that AQP9 mRNA and protein levels are reduced in human hepatocellular cancer compared to the para-tumor normal liver tissues. AQP9 overexpression in SMMC7721 human hepatoma cell line inhibits cell colony formation and xenograft liver tumor growth. The possible mechanisms are that AQP9 reduces PI3K and P-Akt levels, leading to up-regulation of FOXO1 expression and subsequently cell cycle arrest at the G1 phase and cellular apoptosis. These findings suggest that restoration of AQP9 expression can inhibit development of liver cancer. Future studies shall determine how AQP9 expression is down-regulated and what approaches are clinically feasible to restore AQP9 expression, thus developing novel strategies for the prevention and treatment of HCC.

## MATERIALS AND METHODS

### Human liver specimens

Thirty-four cases of human HCC were included, who were surgically treated in the Departments of Hepatobiliary Surgery at the First and Second Affiliated Hospitals of Chongqing Medical University, Chongqing, China, from October 1, 2012 to March 30, 2013. Immediately after surgery, macroscopic tumor tissues and their para-tumor normal liver tissues (approximately 2 cm away from the tumor margin) were collected. A portion of each tissue was used for protein and RNA extractions, respectively. The remaining tissues were fixed with 4% paraformaldehyde, embedded in paraffin, and cut into 4 μm-thick sections. All patients signed informed consent forms prior to surgery. None of the patients had received any prior radiotherapy or chemotherapy. This study was approved by the Chongqing Medical University Ethics Committee and was in accordance with the Ethical Principles for Medical Research Involving Human Subjects as formulated in the World Medical Association Declaration of Helsinki.

### Reagents

Human hepatoma cell line SMMC7721 was purchased from the Cell Bank at the Type Culture Collection of the Chinese Academy of Sciences, Shanghai, China. Lentivirus vector expressing AQP9-enhanced green fluorescent protein (eGFP) (named LV-AQP9) and lentivirus vector expressing eGFP (named LV-PWPI) were obtained from Shanghai Genechem Co., Ltd. (Shanghai, China). Annexin V-phycoerythrin (PE) apoptosis detection kit was purchased from Beyotime Institute of Biotechnology (Shanghai, China). Terminal deoxynucleotidyl transferase dUTP nick end labeling (TUNEL) kit was purchased from Roche (Basel, Switzerland). Cell counting kit-8 (CCK8) assay was purchased from Dojindo (Tokyo, Japan). Rabbit anti-human AQP9 antibody was purchased from Abcam (Cambridge, MA, USA). Rabbit anti-human caspase-3, PI3K, proliferating cell nuclear antigen (PCNA), Akt, P-Akt, and forkhead box protein O1 (FOXO1) antibodies were purchased from Cell Signaling Technology (Danvers, MA, USA). Anti-β-actin antibody and horseradish peroxidase-labeled secondary antibodies were purchased from Beijing Zhongshan Golden Bridge Biotechnology Co., Ltd. (Beijing, China).

### Establishment of a hepatoma cell line stably overexpressing AQP9

SMMC7721 cells were cultured in Dulbecco's Modified Eagle Medium with 10% fetal bovine serum (Gibco, Grand Island, NY, USA) in a humidified incubator at 37°C with 5% CO_2_. The cells were either transfected with LV-PWPI vector (expressing eGFP, thus named as eGFP+ cells) or LV-AQP9 vector (expressing AQP9-eGFP fusion protein, thus named as AQP9-eGFP+ cells). Then, the cells were selected with 1 μg/mL puromycin for 4 weeks.

### Animals

Twelve BALB/c male nude mice at 4-6 weeks of age were provided by the Experimental Animal Center of Chongqing Medical University and were housed in a specific-pathogen-free barrier environment. The experimental protocols were reviewed and approved by the Committee on ethics of animal experiments of the Chongqing Medical University. We confirmed that all experiments were performed in accordance with relevant approved guidelines and regulations. All efforts were taken to minimize animal suffering. Approximately 1.2 million eGFP^*+*^ or AQP9-eGFP^*+*^ cells were injected subcutaneously into the left axilla (*n* = 6 mice per cell line). Tumor size was measured using a caliper once every 3 days. Tumor volume was calculated using a formula = 1/2 (long diameter × short diameter^*2*^) (mm^*3*^). The animals were euthanized 5 weeks later and the tumor tissues were collected for histopathological examinations.

### Immunohistochemical (IHC) staining

Human and animal tumor tissue sections were boiled for 20 minutes in citrate buffer for antigen retrieval and blocked for 20 minutes in 3% hydrogen peroxide. The sections were incubated with primary antibodies overnight at 4°C and then with horseradish peroxidase-conjugated secondary antibodies for 20 minutes at 37°C. The sections were then developed with 3, 3′-diaminobenzidine and counter-stained with hematoxylin. Negative controls used non-immune normal IgG instead of the primary antibodies. Positive controls used a previously known positive sample. The results were assessed by two experienced pathologists who were blinded to the grouping of the samples. IHC staining intensity was measured by randomly selecting 3 high-power (×400) fields per section, using Image-Pro Plus software (Media Cybernetics, Rockville, MD, USA).

### TUNEL assay

Animal tumor sections were digested with 0.5% proteinase K at 37°C for 30 minutes. TUNEL assay was performed according to the manufacturer's instructions. Negative controls used fluorescein-labeled dUTP. Six high-power (×400) fields per section were randomly selected and counted to obtain the numbers of TUNEL-positive cells and the total cell number. The percentage of apoptotic cells was calculated by dividing the number of TUNEL-positive cells with the total cell number.

### Western blot analysis

Human HCC, normal liver samples, and cells were homogenized for protein extraction. Equal amount of the proteins was resolved on 12% sodium dodecyl sulfate-polyacrylamide gel electrophoresis and transferred to a polyvinylidene fluoride membrane. The membrane was blocked with 5% non-fat milk or 5% BSA for 1 hour and incubated with primary antibodies overnight at 4°C, followed by incubation with secondary antibodies for 1 hour at 37°C. The membrane was developed using the enhanced chemiluminescence method and imaged with a GEL DOC XR gel imager (Bio-Rad, Hercules, CA, USA). Using Quantity One software (Bio-Rad), the integrated density values of target protein bands were normalized by those of β-actin to obtain relative protein expression levels, except that the integrated density values of P-Akt bands were normalized by those of Akt bands to obtain the ratios of P-Akt/Akt.

### Reverse transcription PCR (RT-PCR)

Total RNA was extracted from human tissues or cells using TRIzol. One μg of RNA was used for reverse transcription using the TaKaRa two-step method. The resulting cDNA was used for real-time quantitative RT-PCR. Glyceraldehyde 3-phosphate dehydrogenase (GAPDH) was used as the internal control and the results were analyzed using CFX96 Manager software. The primer sequences were: GAPDH forward 5′-CGATGCCCTGAGGCTCTTT-3′and reverse 5′-TGGATGCCACAGGATTCCAT-3′; AQP9 forward 5′-AAATAAACCTCCTTGGCCTGA-3′ and reverse 5′-GCAACAAACATCACCACACC-3′.

### Apoptosis analysis

The cells were incubated in 190 μL of Annexin V-PE binding solution, 5 μL of Annexin V-PE, and 5 μL of 7-amino-actinomycin D (7-AAD, BioLegend, San Diego, CA, USA) in the dark at room temperature for 20 minutes and analyzed using flow cytometry to measure the percentage of apoptotic cells in the window of 7-AAD/Annexin V-PE double stained population.

### Cell cycle analysis

The cells were fixed in pre-cooled 75% ethanol for 12 hours and incubated in 500 μL of PBS with propidium iodide and RNAse A at final concentrations of 50 μg/mL for 30 minutes at 37°C. Flow cytometry analysis was performed to determine the percentage of cells in the G1, S, and G2/M phase of cell cycle.

### Colony formation assay

The cells were seeded in triplicate wells per group in 6-well plates and the cells were plated in a 1 ml volume of cells at a density of 1 × 10^3^ per well for 4 weeks. The cell colonies were fixed with 4% paraformaldehyde for 30 min and stained with Giemsa staining solution. The number of colonies per well was counted and colony formation efficiency was calculated as the number of colonies divided by the number of cells seeded × 100%.

### Cell counting kit-8 assay

The cells were seeded in sextuplicate wells per group in 96-well plates at 5 × 10^3^ cells and were grown for 24, 48, 72, 96 h after transfection according to the manufacturer's instructions. The number of living cells was calculated by every 24 h for four days using a cell counting kit-8 (CCK8) assay. A CCK8 solution was added to each well and the cells were incubated for an additional 2 h. The absorbance at a wavelength of 450 nm was tested using a microplate reader.

### Statistical analysis

Statistical Package for the Social Sciences (SPSS) 19.0 (SPSS Inc, USA) was used to analyze the results. The data was presented as mean ± standard error of the mean (the error bar). The relative risk of AQP9 was assessed by Chi-square test. Multiple sets of data were compared using one-way analysis of variance (ANOVA). The paired tumor and normal tissue data were compared using paired Student's *t* tests (two-sided). *P* value < 0.05 was considered statistically significant.

## SUPPLEMENTARY MATERIALS


