# Supplementary material for: Aquaporin 9 inhibits hepatocellular carcinoma through up-regulating FOXO1 expression
Source: Oncotarget. 2016 Jun 17;7(28):44161–70. doi: 10.18632/oncotarget.10143 (PMC5190086; doi:10.18632/oncotarget.10143)
Supplement: Supplementary file 1 [file oncotarget-07-44161-s001.pdf]

Aquaporin 9 inhibits hepatocellular carcinoma through up-regulating FOXO1 expression

Supplementary Materials

Supplementary Table S1: Associations between AQP9 expression levels (liver cancer/ the para-tumor normal liver tissues) and clinicopathologic features of HCC patients (*n* = 34)

Characteristics	Numbers	Expression level of AQP9 protein		*P* values	χ2
		Normal	Low		
**All cases**	34	8	26		
**Gender**					
Male	29	7(24.14%)	22(75.86%)	0.840	0.041
Female	5	1(20.00%)	4(80.00%)		
**Age(y)**					
<45	8	3(37.50%)	5(62.50%)	0.287	1.135
≥45	26	5(19.23%)	21(80.77%)		
**Tumor size**					
<2cm	7	2(28.57%)	5(71.43%)	0.724	0.125
≥2cm	27	6(22.22%)	21(77.78%)		
**Tumor number**					
Single	29	7(24.14%)	22(75.86%)	0.840	0.041
Multiple	5	1(20.00%)	4(80.00%)		
**Tumor grade**					
Moderate or High	24	8(33.33%)	16(66.67%)	0.037*	4.359
Low	10	0(0.00%)	10(100.00%)		
**Tumor stage**					
I ~II	23	8(34.78%)	15(65.22%)	0.025*	5.003
III ~ IV	11	0(0.00%)	11(100.00%)		
**PVTT**					
Presence	5	0(0.00%)	5(100.00%)	0.179	1.804
Absence	29	8(27.59%)	21(72.41%)		
**LM**					
Presence	3	0(0.00%)	3(100.00%)	0.314	1.012
Absence	31	8(25.81%)	23(74.19%)		
**TM**					
Presence	12	0(0.00%)	12(100.00%)	0.017*	5.706
Absence	22	8(36.36%)	14(63.64%)		
**HBsAg**					
Positive	30	7(23.33%)	23(76.67%)	0.941	0.005
Negative	4	1(25.00%)	3(75.00%)		
**AFP (μg/L)**					
< 400	27	8(29.63%)	19(70.37%)	0.100	2.712
≥ 400	7	0(0.00%)	7(100.00%)		
**Liver cirrhosis**					
Presence	12	3(25.00%)	9(75.00%)	0.881	0.022
Absence	22	5(22.73%)	17(77.27%)		

PVTT: portal vein tumor thrombosis; LM: lymphatic metastasis TM: tumor metastasis; AFP: alpha-fetoprotein; *$P < 0.05$.
